# Development and validation of a risk nomogram model for predicting pulmonary hypertension in patients with stage 3–5 chronic kidney disease

**DOI:** 10.1007/s11255-022-03431-x

**Published:** 2022-12-23

**Authors:** Yue Hu, Xiaotong Wang, Shengjue Xiao, Huimin Wu, Chunyan Huan, Tao Xu, Minjia Guo, Ailin Liu, Xiaoyao Jiang, Jia Wang, Hong Zhu, Defeng Pan

**Affiliations:** 1 Beijing Hospital of Traditional Chinese Medicine, Capital Medical University, Intensive Care Unit Department, No. 23, Mei Shu Guan Hou Jie, Beijing, 100010 Dongcheng China; 2 Department of Cardiology, The Affiliated Hospital of Xuzhou Medical University, Xuzhou, 221004 Jiangsu China; 3 Department of Cardiology, School of Medicine, Zhongda Hospital, Southeast University, 87 Dingjiaqiao, Nanjing, 210009 Jiangsu China; 4 Department of Nephrology, The Affiliated Hospital of Xuzhou Medical University, Xuzhou, 221004 Jiangsu China

**Keywords:** Chronic kidney disease, Pulmonary arterial hypertension, Nomogram, Prediction model

## Abstract

**Objectives:**

The occurrence of pulmonary arterial hypertension (PAH) can greatly affect the prognosis of patients with chronic kidney disease (CKD). We aimed to construct a nomogram to predict the probability of PAH development in patients with stage 3–5 CKD to guide early intervention and to improve prognosis.

**Methods:**

From August 2018 to December 2021, we collected the data of 1258 patients with stage 3–5 CKD hospitalized at the Affiliated Hospital of Xuzhou Medical University as a training set and 389 patients hospitalized at Zhongda Hospital as a validation set. These patients were divided into PAH and N-PAH groups with pulmonary arterial systolic pressure ≥ 35 mmHg as the cutoff. The results of univariate and multivariate logistic regression analyses were used to establish the nomogram. Then, areas under the receiver operating characteristic curve (AUC-ROCs), a calibration plot, and decision curve analysis (DCA) were used to validate the nomogram.

**Results:**

The nomogram included nine variables: age, diabetes mellitus, hemoglobin, platelet count, serum creatinine, left ventricular end-diastolic diameter, left atrial diameter, main pulmonary artery diameter and left ventricular ejection fraction. The AUC-ROCs of the training set and validation set were 0.801 (95% confidence interval (CI) 0.771–0.830) and 0.760 (95% CI 0.699–0.818), respectively, which showed good discriminative ability of the nomogram. The calibration diagram showed good agreement between the predicted and observed results. DCA also demonstrated that the nomogram could be clinically useful.

**Conclusion:**

The evaluation of the nomogram model for predicting PAH in patients with CKD based on risk factors showed its ideal efficacy. Thus, the nomogram can be used to screen for patients at high risk for PAH and has guiding value for the subsequent formulation of prevention strategies and clinical treatment.

**Supplementary Information:**

The online version contains supplementary material available at 10.1007/s11255-022-03431-x.

## Background

Chronic kidney disease (CKD) is a life-threatening chronic disease which mainly manifests as kidney function impairment, and it is characterized by irreversible renal dysfunction and loss of homeostasis. The International Organization of Nephrology 2012 “Kidney Disease: Improving Global Outcomes” (KDIGO) produced CKD guidelines and proposed guiding recommendations for the definition, staging, diagnosis, treatment, and prevention of the disease [1]. CKD is a worldwide health problem and one of the leading causes of morbidity and mortality, affecting more than 10% of the world's population [2, 3]. With economic development and the accompanying changes in lifestyle and diet, the incidence of some nutritional metabolic diseases, such as hypertension, diabetes mellitus (DM), and obesity, has increased significantly. These metabolic diseases lead to an increase in the incidence of CKD [4, 5]. According to an epidemiological study, CKD replaced malnutrition and infection as the leading causes of mortality during the twentieth century [6].

Pulmonary arterial hypertension (PAH) is a small pulmonary artery disease characterized by vascular remodeling, and it can eventually lead to heart failure and death by increasing resistance in blood vessels in the lungs [7]. The gold standard for PAH diagnosis is right heart catheterization, but it is an invasive procedure with high risk. Therefore, it is not recommended for clinical monitoring [8]. Cardiac color Doppler ultrasound is a convenient and noninvasive examination method that can efficiently determine whether the heart tissue is abnormal in terms of anatomy and function. Therefore, it has been unanimously recognized by the medical community [9]. The early clinical symptoms of PAH are not atypical, so the early diagnosis rate is low.

PAH is common in patients with CKD and was found to occur in 56% of patients [10]. Investigations have shown that the incidence of PAH is related to the type of dialysis selected by patients with end-stage renal disease: the rate of PAH was 18.8–68.8% in maintenance hemodialysis patients [11] and 12–42% in peritoneal dialysis patients [12, 13]. However, the pathogenesis of PAH in patients with CKD has not been completely elucidated, and it may be related to anemia, diabetes mellitus, left ventricular structure and function, and dialysis mode [14–16]. At present, in patients with CKD, especially in patients with stage 3–5 chronic renal failure, the independent risk factors and the specific incidence of pulmonary hypertension are still unknown.

A nomogram is a simple, personalized visualization tool that has been widely used in diagnostic and prognostic determinations for cancer patients [17]. A study has shown that nomograms potentially represent an ideal model for predicting the prognosis of CKD patients [18]. However, no nomogram has been used to predict the risk of pulmonary hypertension in patients with CKD. In this study, we aimed to construct a nomogram to predict the probability of developing PAH in patients with stage 3–5 CKD to guide clinical diagnosis and early intervention and to improve prognosis.

## Materials and methods

### Study population and design

This retrospective study was based on data within an electronic medical record system. Patients who were hospitalized in the Department of Nephrology at the Affiliated Hospital of Xuzhou Medical University and Zhongda Hospital affiliated with Southeast University from August 2018 to December 2021 and diagnosed with stage 3–5 CKD according to the 2012 KDIGO guidelines were included in this study [1]. This study was approved by the Medical Research Ethics Committee of the Affiliated Hospital of Xuzhou Medical University (approval number XYFY2022-KL093-01). Because the study was retrospective, the review committee waived the requirement for written informed consent.

Inclusion criteria were as follows: (i) referring to the 2012 KDIGO CKD guideline*,* a diagnosis of stage 3–5 CKD; (ii) age ≥ 18 years, and for patients in the dialysis group, a maintenance hemodialysis (MHD) or peritoneal dialysis (PD) duration of at least 3 months; (iii) complete data for laboratory tests and examination indicators; and (iv) for patients in the dialysis group, the attainment of the dialysis adequacy standard before cardiac color Doppler examination. The exclusion criteria were as follows: (i) loss to follow-up; (ii) an MHD or PD duration < 3 months; (iii) MHD combined with PD; (iv) a history of cancer; (v) severe liver insufficiency; (vi) severe lung disease; and (vii) other reasons. Patients who met the inclusion criteria were examined by Doppler echocardiography. According to their pulmonary arterial pressure, they were divided into a PAH group and an N-PAH group. After admission, all patients with stage 3–5 CKD were received routine renoprotective therapy.

### Echocardiographic detection

The clinical endpoint was defined as the occurrence of PAH in patients with stage 3–5 CKD, and pulmonary arterial systolic pressure (PASP) was calculated according to Bernoulli's formula after tricuspid regurgitation velocity was measured by an experienced color echocardiologist using a Philips EPIQ 7C color Doppler echocardiograph with a probe frequency of 1–5 MHz. According to the American Society of Echocardiography Guidelines for the Evaluation of Adult Right Heart Echocardiography, PASP > 35 mmHg was defined as PAH [19].

### Predictor variables

Relevant literature was reviewed, and the factors that initially affected the grouping of patients included sex, age, body mass index (BMI), New York Heart Association (NYHA) function classification, smoking history, alcohol consumption history, hypertension, DM, cerebral infarction and coronary heart disease, dialysis way, etc. Laboratory indicators of patients in both groups, such as low density lipoprotein cholesterol (LDL-C), total cholesterol (TC), uric acid (UA), and serum albumin level, and Doppler echocardiography indicators, such as left ventricular ejection fraction (LVEF), left atrial diameter (LAD), left ventricular posterior wall diameter (LVPWD), left ventricular end diastolic diameter (LVDd), main pulmonary artery diameter (MPAD), and right ventricle diameter (RVD), were evaluated. For all patients, blood samples were obtained within 24 h of admission and were used for the determination of the above indicators, and patients underwent echocardiography within 48 h of admission. All patients were discharged, and follow-up records, regular outpatient visits, and telephone follow-up were established.

### Statistical analysis

In this study, SPSS 22.0 statistical software was used to analyze the data. The Shapiro‒Wilk test and Levene test were used to evaluate the normality and homogeneity of measurement data variance. The measurement data conforming to a normal distribution are represented by the mean ± standard deviation, while the measurement data conforming to a nonnormal distribution are represented by the median (M) and interquartile range (M P25, P75). Count data are expressed as frequencies or percentages (%). For the measurement data conforming to a normal distribution, two independent-sample *T* tests were used for intergroup comparison. A nonparametric test (Mann‒Whitney *U* test) was used for between-group comparisons of the measurement data with a nonnormal distribution. The chi-square test or Fisher's exact probability method was used for between-group comparisons of the count data. Univariate and multivariate logistic regression methods were used to establish a predictive model represented by a nomogram. Variables with *P* < 0.05 in univariate analysis were included in the multivariate regression analysis. Based on the results of multivariate analysis, we established the nomogram. Then, we used the areas under the ROC and calibration plots to verify the nomogram. In addition, we also used a calibration plot to verify the model internally and DCA to determine the clinical usefulness of the nomogram.

## Results

### Baseline patient characteristics in the training set

**Fig. 1:**
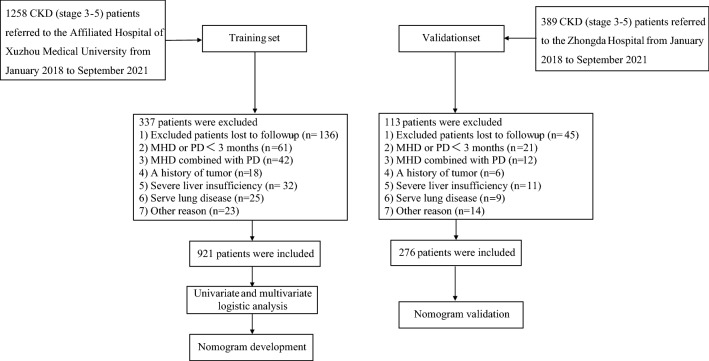
Flow chart of inclusion and exclusion process of patients with CKD (stage 3–5). *CKD* chronic kidney disease, *MHD* maintenance hemodialysis, *PD* peritoneal dialysis, *PAH* pulmonary arterial hypertension

Between August 2018 and September 2021, a total of 1258 CKD (stage 3–5) patients were referred to the affiliated Hospital of Xuzhou Medical University, and the data of 921 patients were selected as the training set for analysis according to the exclusion criteria. At the same time, we also selected 389 patients with CKD (stage 3–5) hospitalized in Zhongda Hospital affiliated with Southeast University and finally included the data of 276 patients as the validation set for analysis. The specific process is shown in Fig. 1.

### Comparison of the general data of patients in the training set

Compared with the N-PAH group, patients in the PAH group were older and had a lower BMI. The histories of hypertension and DM were also significantly different between the two groups (*P* < 0.05). In other general data, there was no significant difference between the two groups. The general data characteristics of the PAH group and N-PAH group are shown in Table 1.

**Table 1:** Comparison of the general conditions of patients in the N-PAH group and PAH group in training set

	N-PAH (*n* = 585)	PAH (*n* = 336)	*P* value
Age, years	54.75 ± 15.22	61.22 ± 14.47	< 0.001
Gender (*n*, %)			
Male	368 (63.2%)	214 (36.8%)	0.812
Female	217 (64.0%)	122 (36.0%)	
Smoking (*n*, %)			
No	443 (64.7%)	242 (35.3%)	0.215
Yes	142 (60.2%)	94 (39.8%)	
Drinking (*n*, %)			
No	474 (64.1%)	265 (35.9%)	0.429
Yes	111 (61.0%)	71 (39.0%)	
Hypertension (*n*, %)			
No	94 (71.2%)	38 (28.8%)	0.047
Yes	491 (62.2%)	298 (37.8%)	
Diabetes mellitus (*n*, %)			< 0.001
No	499 (85.3%)	242 (72.0%)	
Yes	86 (14.7%)	94 (28.0%)	
History of CHD (*n*, %)			
No	546 (63.9%)	308 (36.1%)	0.349
Yes	39 (58.2%)	28 (41.8%)	
History of cerebral infarction (*n*, %)			0.475
No	521 (63.9%)	294 (36.1%)	
Yes	64 (60.4%)	42 (39.6%)	
Lung infection during dialysis			0.888
No	510 (63.4%)	294 (36.6%)	
Yes	75 (64.1%)	42 (35.9%)	
Protopathic			
Chronic glomerulonephritis	192 (61.3%)	121 (38.7%)	0.068
Diabetic nephropathy	187 (62.5%)	112 (37.5%)	
Hypertensive nephropathy	112 (61.5%)	70 (38.5%)	
Other reasons	94 (74.0%)	33 (26.0%)	
Dialysis way (*n*, %)			0.690
Without dialysis	370 (64.6%)	203 (35.4%)	
MHD	149 (61.6%)	93 (38.4%)	
PD	66 (62.3%)	40 (37.7%)	
BMI (kg/m^2^)	23.60 (21.91, 25.34)	23.22 (21.98, 24.30)	0.013

*CHD* coronary heart disease, *MHD* Maintenance hemodialysis, *PD* peritoneal dialysis, *BMI* body mass index;

### Comparison of the laboratory results of patients in the training set

Compared with the N-PAH group, the red blood cell (RBC) count, hemoglobin (Hb), and platelet (PLT) count were lower and serum creatinine (Scr), fasting blood glucose (FBG), and parathyroid hormone (PTH) were higher in the PAH group, and the differences were statistically significant (*P* < 0.05). Specific results are shown in Table 2.

**Table 2:** Comparison of laboratory results of patients in training set

	N-PAH (*n* = 585)	PAH (*n* = 336)	*P* value
WBC (10^9^/L)	6.30 (5.00, 7.80)	6.30 (5.20, 7.90)	0.317
RBC (10^12^/L)	3.65 (3.14, 4.01)	3.60 (3.14, 3.86)	0.045
HB (g/L)	94.00 (86.00, 105.00)	85.00 (75.00, 100.75)	< 0.001
PLT (10^9^/L)	208.00 (163.00, 254.00)	179.00 (145.25, 231.00)	< 0.001
hs-CRP (mg/L)	8.90 (4.22, 14.03)	7.89 (3.40, 12.78)	0.069
ALB (g/L)	35.00 (28.00, 43.00)	34.00 (29.00, 40.00)	0.282
UREA (mmol/L)	22.34 (11.41, 44.45)	20.44 (13.33, 33.06)	0.231
SCr (umol/L)	614.00 (367.00, 792.00)	671.50 (519.25, 833.00)	< 0.001
UA (umol/L)	455.00 (350.50, 590.00)	443.00 (356.00, 520.75)	0.069
eGFR (ml/min)	29.26 (19.39, 40.55)	29.42 (18.34, 39.26)	0.089
FBG (mmol/L)	4.63 (3.49, 6.11)	4.86 (4.18, 6.38)	< 0.001
TC (mmol/L)	4.20 (3.44, 5.79)	4.35 (3.46, 6.01)	0.284
TG (mmol/L)	2.38 (1.34, 4.32)	2.46 (1.37, 4.48)	0.67
HDL-C (mmol/L)	1.20 (0.91, 1.59)	1.16 (0.89, 1.51)	0.218
LDL-C (mmol/L)	2.58 (1.76, 3.45)	2.66 (1.86, 3.41)	0.66
Lp (a) (mg/L)	406.00 (266.50, 555.50)	414.00 (275.25, 566.75)	0.449
K (mmol/L)	4.47 (3.75, 5.11)	4.55 (3.79, 5.29)	0.097
Na (mmol/L)	139.59 (136.70, 142.52)	140.20 (136.22, 143.06)	0.459
Cl (mmol/L)	100.63 (96.25, 104.50)	101.10 (96.92, 105.59)	0.1
Mg (mmol/L)	0.88 (0.64, 1.23)	0.92 (0.61, 1.32)	0.335
Ca (mmol/L)	2.23 (1.60, 2.60)	2.17 (1.47, 2.55)	0.079
P (mmol/L)	1.43 (1.14, 1.77)	1.46 (1.18, 1.80)	0.184
LDH (U/L)	246.00 (172.50, 330.00)	261.00 (173.25, 342.50)	0.117
CK (U/L)	98 (48.00, 159.00)	87.00 (52.00, 145.00)	0.414
CK-MB (U/L)	2.63 (1.47, 5.78)	2.12 (1.35, 4.67)	0.051
AT-III (%)	84.00 (68.00, 103.00)	84.00 (68.00, 101.75)	0.889
PT-INR	1.04 (0.81, 1.31)	1.06 (0.83, 1.34)	0.446
APTT (sec)	28.10 (24.35, 35.95)	28.90 (24.73, 38.25)	0.127
D-Di (ug/ml)	3.15 (1.90, 5.19)	3.16 (1.92, 5.67)	0.552
FT3 (pmol/L)	3.26 (2.28, 4.44)	3.24 (2.05, 4.33)	0.504
FT4 (pmol/L)	14.92 (11.52, 17.86)	15.06 (11.48, 18.20)	0.924
TSH (mIU/L)	3.05 (1.68, 6.59)	2.80 (1.79, 6.05)	0.375
Ferritin (ng/mL)	267.63 (96.63, 564.06)	283.76 (111.99, 566.60)	0.605
Folic acid (ng/mL)	5.51 (2.70, 11.05)	5.73 (2.71, 11.67)	0.677
Vitamin B12 (ng/mL)	531.84 (241.00, 1020.76)	475.91 (210.50, 942.17)	0.097
PTH (pg/ml)	210.00 (129.50, 400.00)	237.00 (155.00, 426.00)	0.004

*WBC* white blood cell, *RBC* red blood cell, *HB* hemoglobin, *PLT* platelet, *hs-CRP* high-sensitivity C-reactive protein, *Alb* albumin, *Scr* serum creatinine, *UA* uric acid, *eGFR* estimated glomerular filtration rate, *FBG* fast blood glucose, *TC* total cholesterol, *TG* triglyceride, *HDL-C* high density lipoprotein-cholesterol, *LDL-C* low density lipoprotein-cholesterol, *Lp(a)* Lipoprotein a, *K* potassium, *Na* sodium, *Cl* chlorine, *Mg* magnesium, *Ca* calcium, *P* phosphorus, *LDH* lactic dehydrogenase, *C.K.* creatine kinase, *CKMB* creatine kinase-MB, *AT-III* Antithrombin III activity, *PT-INR* PT-international normalized ratio, *APTT* activated partial thromboplastin time, *d-Di* d-dimer, *FT3* free triiodothyronine, *FT4* free tetraiodothyronine, *TSH* thyroid-stimulating hormone, ferritin folic acid, *PTH* parathyroid hormone

### Comparison of the Doppler echocardiography results of patients in the training set

The LAD, LVDd, LVPWD, RVD, and MPAD in the PAH group were higher than those in the N-PAH group, while LVEF was lower than that in the N-PAH group, and the differences were statistically significant (*P* < 0.05), as shown in Table 3.

**Table 3:** Comparison of Doppler echocardiography results of patients in training set

	N-PAH (*n* = 585)	PAH (*n* = 336)	*P* value
ARD (mm)	30 (28, 33)	31 (29, 33)	0.113
LAD (mm)	41 (36, 47)	45 (39, 50)	< 0.001
IVST (mm)	12 (11, 15)	13 (11, 15)	0.105
LVDd (mm)	50 (45, 56)	53 (46, 58)	< 0.001
LVPWD (mm)	11 (10, 13)	12 (11, 13)	0.009
RVD (mm)	23 (21, 25)	24 (22, 26)	< 0.001
MPAD (mm)	25 (22, 28)	27 (24, 30)	< 0.001
LVEF (%)	58 (54, 63)	53 (49, 60)	< 0.001
FS (%)	31 (23, 37)	30 (22, 36)	0.289

*ARD* aortic root diameter, *LAD* left atrial diameter, *IVST* interventricular septal thickness, *LVDd* Left ventricular end-diastolic diameter, *LVPWD* Left ventricular posterior wall diameter, *RVD* right ventricle diameter, *MPAD* main pulmonary artery diameter, *LVEF* left ventricular ejection fraction, *FS* fraction shortening

### Clinical features of the training and validation sets

To prevent the overfitting of the clinical prediction model in the analysis of influencing factors, we performed an analysis of the differences between the data in the training and validation sets. None of the differences in the features of the training and validation sets were significant, indicating that the dataset was reasonably divided. The basic information of the two groups was comparable, as shown in Supplementary Table S1.

### Predictive nomogram development

As shown in Table 4, univariate and multivariate logistic regression analyses were used to determine the risk factors for PAH. In univariate logistic regression analysis, age, hypertension, DM, Hb, PLT count, Scr, LVDd, LAD, MPAD, and LVEF were identified (*P* < 0.05). Then, we included the above factors in the multivariate analysis, and the independent risk factors for pulmonary hypertension were determined to be age (odds ratio (OR) 1.032; 95% confidence interval (CI) 1.020–1.043), DM (OR 1.942; 95% CI 1.317–2.863), Hb (OR 0.969; 95% CI 0.960–0.978), PLT count (OR 0.995; 95% CI 0.992–0.997), Scr (OR 1.001; 95% CI 1.000–1.002), LVDd (OR 1.043; 95% CI 1.020–1.067), LAD (OR 1.033; 95% CI 1.013–1.054), MPAD (OR 1.098; 95% CI 1.056–1.141) and LVEF (OR 0.946; 95% CI 0.931–0.962).

**Table 4:** Univariate and multivariate logistic analysis for the development of PAH

Variables	Univariate analysis	*P *value	Multivariate analysis	*P* value
	OR (95% CI)		OR (95% CI)	
Age (years)	1.030 (1.020,1.040)	< 0.001	1.032 (1.020, 1.043)	< 0.001
Hypertension	1.501 (1.003, 2.247)	0.048	1.316 (0.834, 2.077)	0.239
DM	2.254 (1.620, 3.136)	< 0.001	1.942 (1.317, 2.863)	0.001
Hb (g/L)	0.971 (0.963, 0.979)	< 0.001	0.969 (0.960, 0.978)	< 0.001
PLT (10^12^/L)	0.994 (0.992, 0.996)	< 0.001	0.995 (0.992, 0.997)	< 0.001
Scr	1.001 (1.001, 1.002)	< 0.001	1.001 (1.000, 1.002)	0.003
UA (umol/L)	0.999 (0.998, 1.000)	0.053		
LVDd (mm)	1.047 (1.026, 1.068)	< 0.001	1.043 (1.020, 1.067)	< 0.001
LAD (mm)	1.048 (1.031, 1.066)	< 0.001	1.033 (1.013, 1.054)	0.001
MPAD (mm)	1.112 (1.075, 1.151)	< 0.001	1.098 (1.056, 1.141)	< 0.001
LVEF (%)	0.946 (0.933, 0.960)	< 0.001	0.946 (0.931, 0.962)	< 0.001

*DM* diabetes mellitus, *RBC* red blood cell, *Hb* hemoglobin, *PLT* platelet, *Scr* serum creatinine, *UA* uric acid, *PTH* parathyroid hormone, *LVDd* left ventricular end diastolic diameter, *LAD* left atrial diameter, *LVPWD* left ventricular posterior wall diameter, *MPAD* main pulmonary artery diameter, *LVEF* left ventricular ejection fraction, *RVD* right ventricle diameter, *OR* odds ratio, *CI* confidence interval

**Fig. 2:**
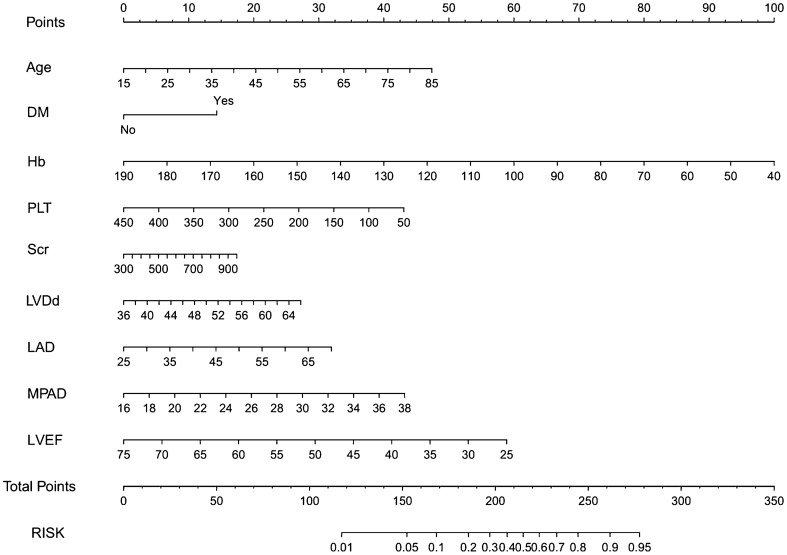
Nomogram used for predicting PAH in patients with CKD. *DM* diabetes mellitus, *Hb* hemoglobin, *PLT* platelet, *Scr* serum creatinine, *LAD* left atrial diameter, *MPAD* main pulmonary artery diameter, *LVEF* left ventricular ejection fraction, *RVD* right ventricle diameter

According to the results of multivariate logistic regression analysis, a nomogram was drawn to predict the occurrence of pulmonary hypertension in patients with stage 3–5 CKD, as shown in Fig. 2. The risk of PAH in patients with stage 3–5 CKD was estimated by calculating the scores corresponding to each risk factor (age, DM, Hb, PLT count, Scr, LVDd, LAD, MPAD, LVEF).

### Validation of the nomogram

**Fig. 3:**
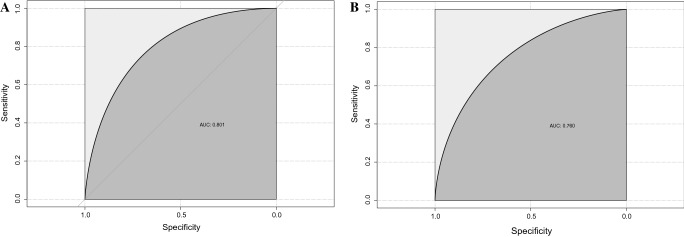
ROC curves of clinical prediction models were drawn based on the data of training set (**A**) and validation set (**B**)

**Fig. 4:**
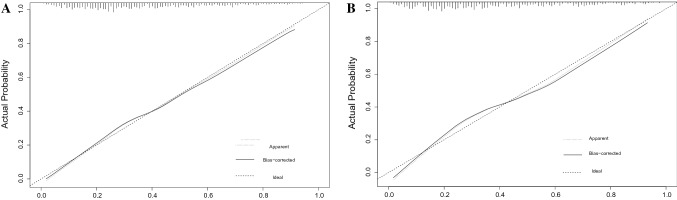
Calibration curve of the nomogram on the data of training set (**A**) and validation set (**B**)

**Fig. 5:**
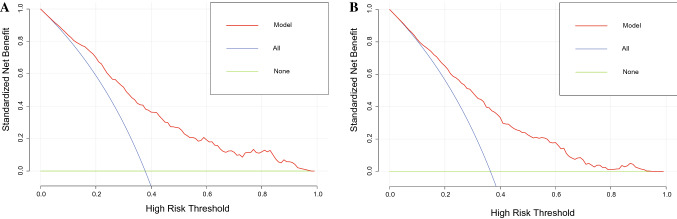
Evaluation of clinical validity of predictive models on the data of training set (**A**) and validation set (**B**)

In the training set, the AUC for predicting PAH in CKD patients was 0.801 (95% confidence interval (CI) 0.771–0.830) (Fig. 3A), and the AUC was 0.760 (95% CI 0.699–0.818) in the validation set (Fig. 3B). This shows that the discriminative ability of this clinical predictive model is very good. Then, we used the bootstrap self-sampling method with *B* = 1000 repetitions and plotted the calibration curves for the training and validation sets of the nomogram. The results showed that the predicted results were in good agreement with the actual results (Fig. 4A, B). In addition, we also used DCA to evaluate the clinical validity of the nomogram (Fig. 5). The results showed that the range of the threshold probability of the nomogram was wide and thus that the model could be clinically useful.

## Discussion

High rates of cardiovascular morbidity and mortality due to CKD place a serious burden on the health care system, and PAH is one of the common complications of CKD [20]. Despite the rapid development of medical treatment, the prognosis and quality of life of CKD patients with PAH are still poor. Therefore, early identification of PAH risk in patients with CKD and early intervention are of great significance. This is the first study to develop a nomogram to predict PAH in patients with CKD, and we found that age, DM, Hb, PLT count, Scr, LVDd, LAD, MPAD, and LVEF were independent risk factors for PAH.

The prevalence of CKD increases with age, and renal function gradually decreases so that 34.0% of people ≥ 65 years old have stage 3 or above CKD [21], which may be related to elderly individuals being more prone to chronic diseases such as hypertension, diabetes, and coronary heart disease. An epidemiological study showed that the prevalence of CKD in elderly individuals over 70 years old reached 47% [22]. Havlucu Y and his team revealed that the age of patients in a PAH group was significantly higher than that in an N-PAH group [23]. Currently, we know that the major risk factors for CKD are diabetes and hypertension [24]: approximately 80–85% of patients with CKD have hypertension, and more than 50% have diabetes [25, 26]. After multivariate logistic analysis, diabetes was identified as a risk factor for PAH in patients with CKD. Torkamani N et al. found that the presence of diabetes and higher HbA1c levels were strongly and independently associated with adverse renal outcomes in patients with CKD who were hospitalized ≥ 2 times. These patients were at high risk for the relatively rapid deterioration of kidney function [27]. DM can cause damage to the pulmonary vascular endothelium and decrease the release of vasodilators, thus aggravating the degree of atherosclerosis, further increasing blood pressure, and eventually leading to high pressure load in the right ventricle and increased pulmonary vascular resistance, resulting in PAH [28, 29]. Agarwal R et al. found that ambulatory systolic blood pressure (BP) was strongly associated with the progression of chronic kidney disease (CKD) and was an independent predictor of ESRD [30]. We found that hypertension was significant in univariate analysis (*P* = 0.031) but not in multivariate analysis (*P* = 0.641). Nevertheless, hypertension may have value for the prediction of PAH in patients with CKD, which can be further explored in future studies.

Regarding laboratory variables, we found that serum creatinine, hemoglobin, and the platelet count were independent risk factors for PAH in patients with CKD. Serum creatinine is a routine biomarker of CKD, which can lead to the late diagnosis of CKD [31]. A 2019 Korean study showed that low Hb levels and anemia were risk factors for ESRD incidence in the general population and CKD progression to ESRD [32]. The main features of PAH include pulmonary vasoconstriction, pulmonary vascular remodeling, and in situ thrombosis. PAH caused by anemia in patients with CKD may be related to the following mechanisms. Low Hb levels reduce the ability of RBCs to carry oxygen, resulting in hypoxemia, which leads to a corresponding increase in heart rate and cardiac output and the constriction of blood vessels in the lungs, eventually leading to PAH [11]. Studies have shown that platelets are involved in the formation and development of PAH and are associated with prognosis through the release of various cytokines that influence inflammatory processes, promote vascular remodeling, and ultimately lead to arterial stenosis [33]. Therefore, active control of serum creatinine, hemoglobin, and the platelet count is of positive significance to the prognosis of patients.

Our study found that in the PAH group, LAD, MPAD, and LVDd were higher than those in the N-PAH group, while LVEF was lower, and the differences were statistically significant. By further logistic analysis, we found that LVEF, left atrial volume (LAD), MPAD, and LVDd were independent risk factors for PAH in patients with CKD. Therefore, changes in cardiac structure and function can affect the occurrence of PAH in patients with CKD. LVEF is an indicator of left ventricular systolic function, and a previous study showed that PAH in patients with CKD is more likely to occur with a low LVEF, which is consistent with our findings [34]. LAD is an indicator of diastolic function [35]. An increase in LAD will increase the pressure of the left atrium, affect pulmonary venous return, and lead to pulmonary circulation congestion, further leading to increased pulmonary arteriole resistance and eventually PAH. Yang et al. confirmed that the occurrence of PAH in patients with CKD is closely related to an enlarged LAD [36]. It is generally believed that the inner diameter of the pulmonary artery in patients with PAH is wider than that in patients without PAH. When PAH occurs in patients with CKD, pulmonary artery pressure increases, and blood in the right ventricle cannot fully return to the left heart. Pulmonary artery vessels also dilate due to changes in pulmonary artery pressure, increasing the MPAD. With the prolongation of dialysis time and the decrease in dialysis adequacy, patients are prone to capacity overload, leading to cardiac systolic and diastolic dysfunction and increasing the risk of PAH. Therefore, to improve the prognosis and quality of life of patients, clinical attention should be given to the changes in cardiac structure and function in patients with CKD and timely treatment.

## Conclusion

We developed a simple nomogram model to predict the risk of pulmonary hypertension in patients with CKD, and the evaluation of this nomogram showed its ideal efficacy. We determined that age, DM, Hb, PLT count, Scr, LVDd, LAD, MPAD, and LVEF were independent risk factors for PAH.

### Limitations

There are some limitations to our study. First, this study is a retrospective study, and the data were obtained from the hospital medical record system. Some risk factors, such as hypervolemia, could not be included in the analysis because the data loss is greater than 30%. Second, when information on the dialysis status of the patients was collected in this study, the specific MHD or PD scheme of the patients was not analyzed in detail; therefore, further research is needed. Third, we did not follow up the CKD patients with PAH after discharge; therefore, information including drug use and all-cause mortality was not obtained.

## Supplementary Information

Below is the link to the electronic supplementary material.

Supplementary file1 (DOCX 31 KB)

Supplementary file2 (XLSX 322 KB)

## Data Availability

The raw data supporting the conclusions of this article will be made available by the authors, without undue reservation.
