# Supplementary material for: Development and validation of a risk nomogram model for predicting pulmonary hypertension in patients with stage 3–5 chronic kidney disease
Source: Int Urol Nephrol. 2022 Dec 23;55(5):1353–63. doi: 10.1007/s11255-022-03431-x (PMC10105676; doi:10.1007/s11255-022-03431-x)
Supplement: Supplementary file 1 — Supplementary file1 (DOCX 31 KB) [file 11255_2022_3431_MOESM1_ESM.docx]

| Table S1. Clinical features of training sets and validation sets. | | | |
| --- | --- | --- | --- |
| Variables | Training (n =921) | Validation (n =276) | *P*-Value |
| Age, years | 57.11±15.264 | 57.5±15.911 | 0.714 |
| Gender (n,%) |  |  | 0.555 |
| Male | 582 (63.2%) | 169 (61.2%) |  |
| Female | 339 (36.8%) | 107 (38.8%) |  |
| Smoking (n,%) |  |  | 0.784 |
| No | 685 (74.4%) | 203 (73.6%) |  |
| Yes | 236 (25.6%) | 73 (26.4%) |  |
| Drinking (n,%) |  |  | 0.557 |
| No | 739 (80.2%) | 217 (78.6%) |  |
| Yes | 182 (19.8%) | 59 (21.4%) |  |
| Hypertension (n,%) |  |  | 0.315 |
| No | 132 (14.3%) | 33 (12.0%) |  |
| Yes | 789 (85.7%) | 243 (88.0%) |  |
| Diabetes mellitus (n,%) |  |  | 0.888 |
| No | 741 (80.5%) | 221 (80.1%) |  |
| Yes | 180 (19.5%) | 55 (19.9%) |  |
| History of CHD (n,%) |  |  | 0.243 |
| No | 854 (92.7%) | 250 (90.6%) |  |
| Yes | 67 (7.3%) | 26 (9.4%) |  |
| History of cerebral infarction (n,%) |  |  | 0.528 |
| No | 815 (88.5%) | 248 (89.9%) |  |
| Yes | 106 (11.5%) | 28 (10.1%) |  |
| Lung infection during dialysis |  |  | 0.328 |
| No | 804 (87.3%) | 247 (89.5%) |  |
| Yes | 117 (12.7%) | 29 (10.5%) |  |
| Protopathy |  |  | 0.967 |
| Chronic glomerulonephritis | 313 (34.0%) | 91 (33.0%) |  |
| Diabetic nephropathy | 299 (32.5%) | 94 (34.1%) |  |
| Hypertensive nephropathy | 182 (19.8%) | 53 (19.2%) |  |
| Other reasons | 127 (13.8%) | 38 (13.8%) |  |
| Dialysis way (n,%) |  |  |  |
| Without dialysis | 573 (62.2%) | 175 (63.4%) | 0.685 |
| MHD | 242 (26.3%) | 66 (23.9%) |  |
| PD | 106 (11.5%) | 35 (12.7%) |  |
| BMI (kg/m^2^) | 23.40 (21.96,24.91) | 23.40 (22.13,24.83) | 0.891 |
| WBC (10^9/L) | 6.30 (5.05,7.85) | 6.30 (5.00,8.00) | 0.788 |
| RBC (10^12/L) | 3.63 (3.14,3.92) | 3.64 (3.14,3.96) | 0.824 |
| Hb (g/L) | 92.00 (82.00,103.00) | 91.00 (82.00,105.00) | 0.905 |
| PLT (10^9/L) | 198.00 (154.50,243.00) | 200.00 (153.25,240.75) | 0.704 |
| hs-CRP, mg/L | 8.49 (4.00,13.53) | 8.55 (3.61,14.68) | 0.744 |
| ALB, g/L | 35.00 (28.00,41.00) | 35.00 (28.00,40.00) | 0.473 |
| UREA（mmol/L） | 21.20 (12.11,39.23) | 19.24 (11.73,44.31) | 0.609 |
| SCr (umol/L) | 620.00 (408.50,820.00) | 616.00 (497.00,792.00) | 0.929 |
| UA (umol/L) | 448.00 (354.00,569.50) | 428.50 (337.50,570.00) | 0.268 |
| eGFR (ml/min) | 29.31 (19.31,39.73) | 30.85 (19.54,40.55) | 0.161 |
| FBG（mmol/L） | 4.71 (3.76,6.15) | 4.70 (3.83,6.11) | 0.891 |
| TC（mmol/L） | 4.28 (3.44,5.83) | 4.27 (3.51,6.07) | 0.570 |
| TG（mmol/L） | 2.41 (1.36,4.34) | 2.35 (1.38,4.26) | 0.987 |
| HDL-C（mmol/L） | 1.18 (0.90,1.56) | 1.23 (0.90,1.54) | 0.797 |
| LDL-C（mmol/L） | 2.60 (1.80,3.43) | 2.60 (1.86,3.53) | 0.396 |
| Lp(a)（mg/L） | 408.00 (272.50,561.50) | 421.00 (271.75,584.00) | 0.749 |
| K（mmol/L） | 4.49 (3.78,5.17) | 4.49 (3.78,5.13) | 0.991 |
| Na（mmol/L） | 139.70 (136.61,142.70) | 139.96 (137.04,142.65) | 0.679 |
| Cl（mmol/L） | 100.83 (96.56,104.92) | 100.90 (97.34,105.18) | 0.545 |
| Mg（mmol/L） | 0.89 (0.63,1.27) | 0.92 (0.64,1.30) | 0.674 |
| Ca（mmol/L） | 2.20 (1.55,2.57) | 2.19 (1.50,2.56) | 0.456 |
| P（mmol/L） | 1.45 (1.16,1.78) | 1.44 (1.16,1.77) | 0.572 |
| LDH（U/L） | 248.00 (173.00,335.00) | 246.00 (167.25,342.75) | 0.983 |
| CK（U/L） | 92.00 (49.50,151.00) | 89.00 (48.25,150.00) | 0.565 |
| CKMB（ng/ml） | 2.43 (1.41,5.43) | 2.54 (1.41,5.33) | 0.815 |
| AT-III（%） | 84.00 (68.00,102.00) | 85.00 (68.25,103.75) | 0.523 |
| PT-INR | 1.05 (0.82,1.32) | 1.04 (0.82,1.33) | 0.924 |
| APTT（sec） | 28.30 (24.40,36.66) | 27.23 (23.88,36.61) | 0.298 |
| D-Di （ug/ml） | 3.16 (1.90,5.27) | 3.31 (1.84,5.67) | 0.575 |
| FT3 （pmol/L） | 3.26 (2.20,4.40) | 3.18 (2.19,4.25) | 0.587 |
| FT4 （pmol/L） | 14.95 (11.50,18.06) | 14.57 (10.91,17.87) | 0.361 |
| TSH （mIU/L） | 2.90 (1.73,6.40) | 2.90 (1.78,6.47) | 0.951 |
| Ferritin（ng/mL） | 273.59 (104.24,565.74) | 290.56 (97.74,578.94) | 0.616 |
| Folic acid（ng/mL） | 5.56 (2.70,11.19) | 5.45 (2.61,11.69) | 0.969 |
| Vitamin_B12（pg/mL） | 506.00 (228.29,987.00) | 582.25 (248.75,1011.72) | 0.401 |
| PTH (pg/ml) | 222.00 (136.50,406.00) | 228.00 (142.00,424.50) | 0.616 |
| ARD (mm) | 31.00 (28.00,33.00) | 31.00 (28.00,33.00) | 0.926 |
| LAD (mm) | 42.00 (37.00,48.00) | 41.00 (36.00,48.00) | 0.894 |
| IVST (mm) | 13.00 (11.00,15.00) | 12.00 (11.00,14.00) | 0.232 |
| LVPWD (mm) | 12.00 (10.00,13.00) | 12.00 (10.00,13.00) | 0.946 |
| LVDd (mm) | 51.00 (45.00,57.00) | 51.00 (45.00,57.00) | 0.766 |
| RVD (mm) | 23.00 (21.00,26.00) | 24.00 (21.00,26.00) | 0.834 |
| MPAD (mm) | 26.00 (23.00,29.00) | 25.00 (23.00,29.00) | 0.357 |
| LVEF (%) | 57.00 (51.50,63.00) | 57.00 (52.00,63.00) | 0.508 |
| FS (%) | 31.00 (23.00,37.00) | 29.50 (22.00,36.00) | 0.387 |

Abbreviations: CHD, coronary heart disease; MHD, Maintenance hemodialysis; PD, [peritoneal](javascript:;) [dialysis](javascript:;); BMI, body mass index; WBC, white blood cell; RBC, red blood cell; HB, hemoglobin; PLT, platelet; hs-CRP, high-sensitivity C-reactive protein; Alb, albumin; Scr, serum creatinine; UA, uric acid; eGFR, estimated glomerular filtration rate; FBG, fast blood glucose; TC, total cholesterol; TG, triglyceride; HDL-C, high density lipoprotein-cholesterol; LDL-C, low density lipoprotein cholesterol; Lp(a), Lipoprotein a; K, potassium; Na, sodium; Cl, chlorine; Mg, magnesium; Ca, calcium; P, phosphorus; LDH, lactic dehydrogenase; CK, creatine kinase; CKMB, creatine kinase-MB; AT-III, Antithrombin III activity; PT-INR, PT-international normalized ratio; APTT, activated partial thromboplastin time; D-Di, D-dimer; FT3, free triiodothyronine; FT4, free tetraiodothyronine; TSH, thyroid stimulating hormone; Ferritin, Folic acid; PTH, Parathyroid Hormone; ARD, Aortic root diameter; LAD, Left atrial diameter; IVST, interventricular septal thickness; LVDd, Left ventricular end-diastolic diameter; LVPWD, Left ventricular posterior wall diameter; RVD, right ventricle diameter; MPAD, Main pulmonary artery diameter; LVEF, Left ventricular ejection fraction; FS, fraction shortening.
